# Clinical Parameters Involved in Skin Tone Perception of Asian Population

**DOI:** 10.1111/jocd.70542

**Published:** 2025-11-20

**Authors:** Ping Wang, Jie Qiu, Juanjuan Chen, Yang Wang, Chengda Ye, Virginie Hourblin, Etienne Huguet, Emmanuelle Mainguene, Frederic Flament

**Affiliations:** ^1^ L'Oréal Research and Innovation Shanghai China; ^2^ L'Oréal Research and Innovation Aulnay‐sous‐Bois France; ^3^ L'Oréal Research and Innovation Rio de Janeiro Brazil; ^4^ L'Oreal Research and Innovation Clichy France

**Keywords:** computer modeling, perception, skin clinical parameters, skin tone, statistics

## Abstract

**Background and Aim:**

Skin tone contributes critically to facial attractiveness; skin aging yellowness has become a predominant aging and skin tone concern in the Asian population. However, the clinical parameters underlying the visual perception of skin yellowness remain elusive. This study explored the relevant clinical parameters and exposome factors influencing the perception of skin yellowness.

**Methods:**

Perceived facial skin yellowness was evaluated through three observation and rating tests involving 170 female participants. Standardized lighting and screen settings ensured consistent evaluations of randomized full face and skin‐tone‐adjusted images. The correlation of yellowness observation and clinical parameters was identified, helping to decode the parameters that determine perception.

**Results:**

The pair test showed that not only b* but also L* and skin diffused reflection determined yellowness perception commonly across age. Rating test on artificial face images confirmed the importance of both b* and L*. A preliminary model on colorimetric parameters was derived: Perceived Yellowness = 0.42b* − 0.2L* + 9.01. Further pair test demonstrated that perceived skin yellowness varied seasonally, suggesting an influence of sun exposure.

**Conclusion:**

This study reveals the correlation of skin yellowness perception and clinical parameters, especially the contribution of relevant colorimetric parameters. From a third‐person perspective, b*, L* and diffused reflection are key determinants of perceived skin yellowness. The observed seasonal variation in yellowness perception indicated a perceivable “seasonal skin yellowing.” The findings provided new insights for developing treatments for skin aging yellowness, photoprotection and modulation of colorimetric parameters (L* and b*) and optical parameter (reflection) are highlighted as important clinical targets.

## 1 Introduction

Skin as the largest and most visible organ of the human body, plays an important role in beauty appearance. Although the preference for aesthetics could be different with multiple social and cultural factors, it is known that the facial skin status of an individual plays an important role in self‐recognition and social interactions. Skin aging is a complex process influenced by multiple intrinsic or chronologic and extrinsic factors, involving changes in skin physiological parameters and clinical characteristics, such as wrinkles, laxity and especially changes in skin tone and pigmentation [1, 2, 3]. With aging, besides similar changes among skin of different ethnic origins, clinical studies showed that Caucasian women reported more signs of moderate and severe facial aging earlier than Asian women [4], while skin tone changes and pigmentary disorders are of greater concern in individuals with skin of color compared with lighter skin [4]. Predominantly, facial skin as a sun‐exposed area of the body, is more prone to photoaging, with more obvious alterations in skin tone and pigmentation [5]. Although Asian skin might be less prone to photodamage [4], it is reported clinically that Asian skin especially skin of North Asian descent, becomes darker and more yellowish with age, which is not the same case as for individuals of other ethnic origins [6].

Skin yellowness or dull yellowness is mentioned a lot in the North Asian population as one of the most predominant aging signs or skin tone concerns, even for the young. Usually, skin aging yellowness appears as a yellowish hue or dull complexion, which may be more visible on the face. Due to the association with beauty standards and cultural beliefs in North Asian countries, the presence of skin aging yellowness has been linked to various factors and implications. Several studies showed that skin tone could play a critical role in social perception; it is demonstrated that both males and females could be sensitive to skin color variations, which could affect the perception of attractiveness, youthfulness and health [6, 7, 8, 9]. Sociopsychological research and consumer studies revealed that the perception of skin tone manifests as a profound internalization of cultural norms, which could influence behavior regarding skincare [10] and lead to strong motivations for brightening product usage [11, 12]. Consistent with the attentional bias framework, individuals exhibiting a stronger fair‐skin preference may engage in more frequent comparative assessments of skin tone, given feelings of dissatisfaction with their own skin tone [13, 14], which may also enhance perceptual sensitivity to skin tone alteration considering the efficacy assessments during skin brightening product usage [15]. It is believed that skin reflects health “inside out,” thus skin aging yellowness can influence people's perceptions of facial attractiveness and health status. Overall yellowish skin may suggest poor health perceptions or an unhealthy lifestyle, which may impact social interactions. Skin aging yellowness may sometimes even cause mental stress to conform to certain expectations in cultural belief, leading to negative emotions and dissatisfaction with appearance.

Skin tone alteration during aging is a complex problem which might be interfered with by numerous factors. It is believed that both environmental factors and lifestyle habits that influence health, such as solar radiation, air pollution, nutritional status, sleep and mental stress, could also be related to skin yellowness complexion [16]. Knowledge of biological changes during aging and biological mechanisms underlying the generation of skin tone might partially explain the dull yellowish complexion. Melanin, as the key contributor to skin pigmentation, has been reported to increase with skin aging [2, 17] especially in skin of color, which could be one of the determinates of skin tone. Besides, yellow components, such as hemoglobin and bilirubin, have also been suspected to be related to skin yellowness [18]. In recent years, more and more studies have addressed the importance of oxidative stress during aging. With aging, the declined oxidation protection system of the human body leads to the accumulation of oxidative stress [19], including oxidatively modified proteins. It has been reported that protein glycation products in facial epidermis correlated with yellow or dull appearance [20], while dermal carbonylation is identified to be correlated with dermal yellowing during skin photoaging [21]. Since blood circulation is believed to be one of the parameters of health status, which is also impacted by the aging process, it has also been linked with yellowish or dull complexion. However, the precise mechanisms behind Asian skin aging yellowness are still not fully understood, especially the correlation of biological changes to perceptual skin changes. Among skin colorimetric parameters, b* value has been recognized as the representative parameter from chromatics to define skin yellowness in facial instrumental analysis in previous studies [8, 9, 12], and recently, brightness and L* value have been mentioned in self‐assessment of parameters for yellowish skin [22]. However, it is still unclear among the physical parameters what the key drivers are that determine the perceived facial skin yellowness from a third‐person perspective and the associated perception thresholds, which is very important for the reveal of the biological mechanism behind and development of cosmetic solutions for yellowness complexion.

In this study, observation and rating tests on Chinese female volunteers were conducted to understand the relevant clinical parameters and the seasonal effect of sun exposure that impact the perception of yellowness of Chinese women. Correlation analysis was applied to all parameters and observation data to identify the significant contributors to perceived skin yellowness.

## 2 Materials and Methods

### 2.1 Historical Facial Image and Clinical Evaluations

The facial images for the below‐described pair tests were taken by Image Table (Orion Concept, France) with standard light in previous skin typology studies. Once zoomed from standardized digital photographs, facial signs were graded by 15 experts, using a referential Skin Aging Atlas of Asian people [23]. Skin colorimetric parameters L*, a* and b* were measured by Chromasphere [24], skin sebum was measured by Sebumeter, while skin reflection‐related parameters C 80% (backscatter light) and P‐C 5% (skin reflection) were measured by the SAMBA face system. Informed consent was obtained prior to the evaluations. The anonymity and confidentiality of all data were maintained throughout the study.

### 2.2 Pair Test

Pair test is a comparative method, designed to understand the perception of an image and the corresponding factors. The 1st pair test aimed to understand the perceived skin yellowness and corresponding clinical factors; 40 healthy Chinese women with normal visual and color resolution acuity, aged 18–35 years old (*N* = 20) and 45–60 years old (*N* = 20), were recruited as observers. They were instructed about the objective of this study, trained on the image evaluation process and signed informed consent as required.

Frontal facial images, taken by Image Table (Orion Concept, France) from a previous typology study, were prepared, including 30 images from 18 to 35 years old females and 35 images from 45 to 60 years old females with skin phototype II–IV. They were randomized for picture‐paired comparison. This test was conducted onsite; all observers visited on a single occasion. To avoid visual age influence and intergroup bias on the perception of skin yellowness, the young and elder group observers were asked to evaluate the images from the same age group as them, separately. All pictures were randomly presented in pairs on the screen side by side at the same time; each observer was shown all the experimental pairs of images in random order, their evaluations were performed under standardized conditions. A HP E233 color display with a resolution of 1920 × 1080 was used; the brightness and color of the screen were calibrated by Spyder X Elite. The observers were sitting directly facing the screen with their eyes 50–60 cm away from it, under standard daylight illumination (D65, at least 450 Lux). When viewing the two full face photographs, a question was asked: Which face looks more yellowish? The observers were asked to click on the facial image that looks more yellowish to them of the two images on the screen. The perception result for each image was presented by the number of clicks. Then the correlation of perception results with existing instrumental evaluation data from a previous typology study was analyzed.

For the 2nd pair test aimed to understand sun exposure impact on yellowness perception, 30 healthy Chinese women with normal visual and color resolution acuity, aged 45–60 years, were recruited as observers. A group of facial pictures of 54 Chinese females from all skin types aged 45–60 years from a previous 1‐year tracking study, including 5 images taken by Image Table (Orion Concept, France) for each image provider during the year, were used as stimuli. The other test conditions were the same as described above.

### 2.3 Quiz Test

200 realistic artificial face images with colorimetric differences were used as stimuli; they were created based on an average face picture, which was generated from the facial images taken by Chromasphere of 200 Chinese women aged from 18 to 30. To avoid the interference of skin texture, there is no skin texture difference on these artificial face images, The images were only modified by adjusting the full skin colorimetric parameters L*, a* and b*.

In this onsite test, all observers visited on a single occasion. 100 volunteers from skin brightening product users, who might pay more attention to skin tone variations [11, 13, 14, 15], aged between 18 and 60 were recruited as observers; they were instructed about the objective of this study, were trained on the image evaluation process and signed informed consent as required. All the stimuli images were presented to observers separately; the evaluations were performed under standardized conditions of lighting, position and screen calibration (by Spyder X Elite). When viewing the full face stimuli images, the observers were asked to attribute a perceptual score on the yellowness level of each image from 1 (not yellow at all) to 9 (very yellow). Then the relation of the scoring results and colorimetric data of each image was analyzed.

### 2.4 Statistics

The observation on images according to the number of clicks (pair test) was analyzed through a correlation test with clinical parameters. The relation of the scoring results (quiz test) on average face images with colorimetric data of each image and the contribution degree of colorimetric parameters was analyzed with a multi‐linear regression model by Scatterplot 3D package in R programming language.

## 3 Results

### 3.1 Correlation of Skin Parameters and Perceptual Yellowness

**FIGURE 1:**
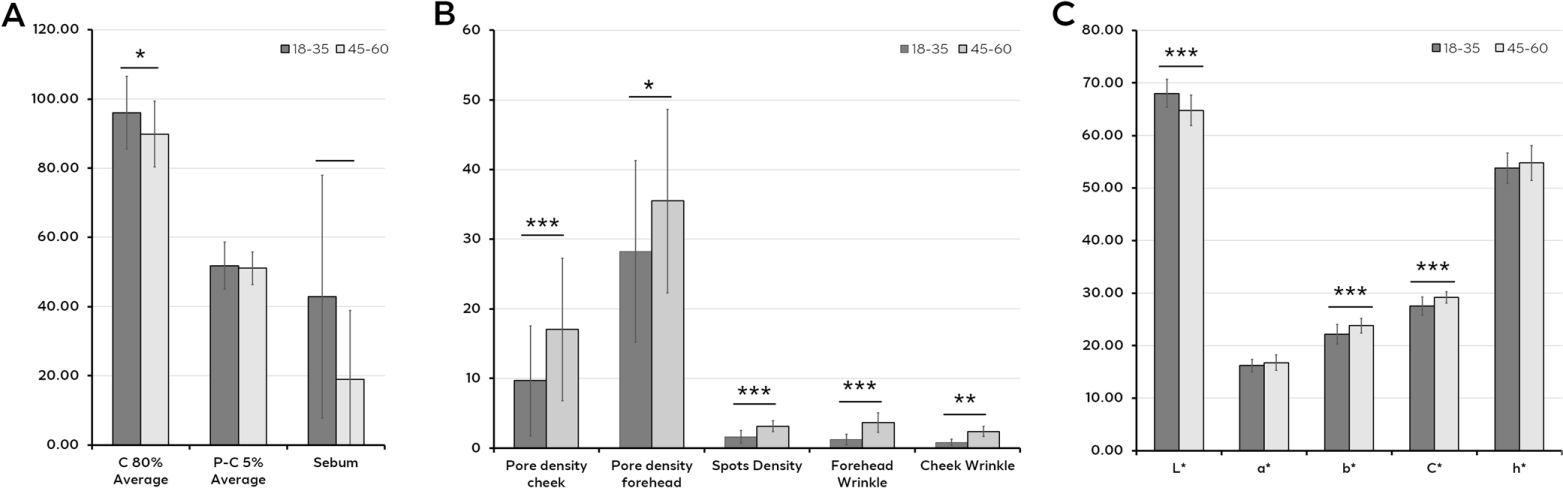
Skin parameters in young and elder age groups. (A) Skin reflection and sebum level. (B) Skin aging signs by clinical evaluation. (C) Colorimetric parameters of skin. Asterisks denote statistically significant differences (*p < 0.05, **p < 0.01, ***p < 0.001).

To understand the perceived skin yellowness and corresponding physical and clinical factors, facial pictures from Chinese female image providers, in two age groups (18–35 years old and 45–60 years old), are put into a pair test. By instrumental analysis of skin parameters of these image providers, compared with the younger group, it is shown in Figure 1 that the elder group has less skin reflection, lower sebum and more obvious full face aging signs, such as wrinkles, pore density, pigmentary disorders. For colorimetric parameters, the elder group showed lower skin lightness and higher b* value (Figure 1), which is consistent with previously published data [6].

**FIGURE 2:**
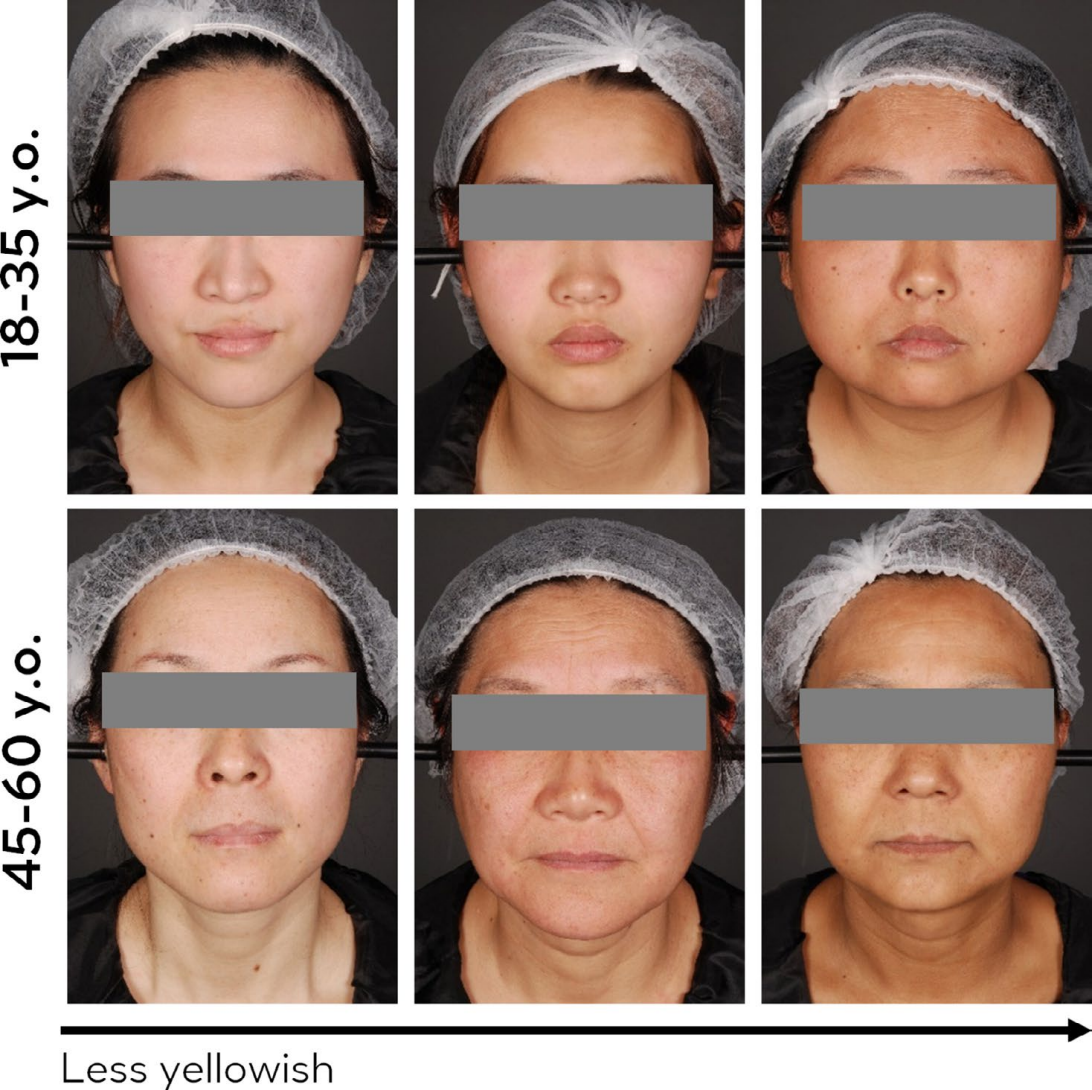
Examples of pictures from young and elder age groups.

To avoid visual age influence and to minimize potential young–elder intergroup bias on perception, the observers were asked to evaluate the images (examples shown in Figure 2) from the same age group as them. Correlation analysis was done to identify the relationship between skin parameters and perceptual skin yellowness. For both age groups, as expected, Chinese females' perception of skin yellowness is positively correlated with b*. However, b* is not the only determinant of perception of skin yellowness; skin lightness (L*) and skin diffused reflection (C 80% average) are significantly negatively correlated with yellowness and dullness perception in both age groups, as shown in Table 1. Besides, in the younger group of people, it is noticed that specular reflection, presented as P‐C 5%, was also shown to be negatively correlated with perceptual skin yellowness. These results suggested that not only colorimetric parameters b* and L* contribute critically to skin yellowness perception, but maybe also skin reflection‐related surface properties or inner‐skin factors could influence the perception of skin yellowness.

**TABLE 1:** Results of correlation analysis of yellowness perception and skin parameters.

Correlation table	C 80%	P‐C 5%	L*	a*	b*	Pore density cheek	Sebum	Spots density
18–35 years old	96.02	51.83	68.02	16.20	22.21	9.68	42.90	1.63
Correlation vs. yellowness perception	−0.71**	−0.56**	−0.61**	0.40	0.52**	0.20	−0.09	0.38
45–60 years old	89.88	51.13	64.80	16.75	23.79	17.03	18.97	3.16
Correlation vs. yellowness perception	−0.58**	−0.32	−0.65**	−0.09	0.52**	0.00	−0.22	0.25

** Indicate significance at 1% level.

### 3.2 Contribution of Skin Colorimetric Parameters to Skin Yellowness Perception

**FIGURE 3:**
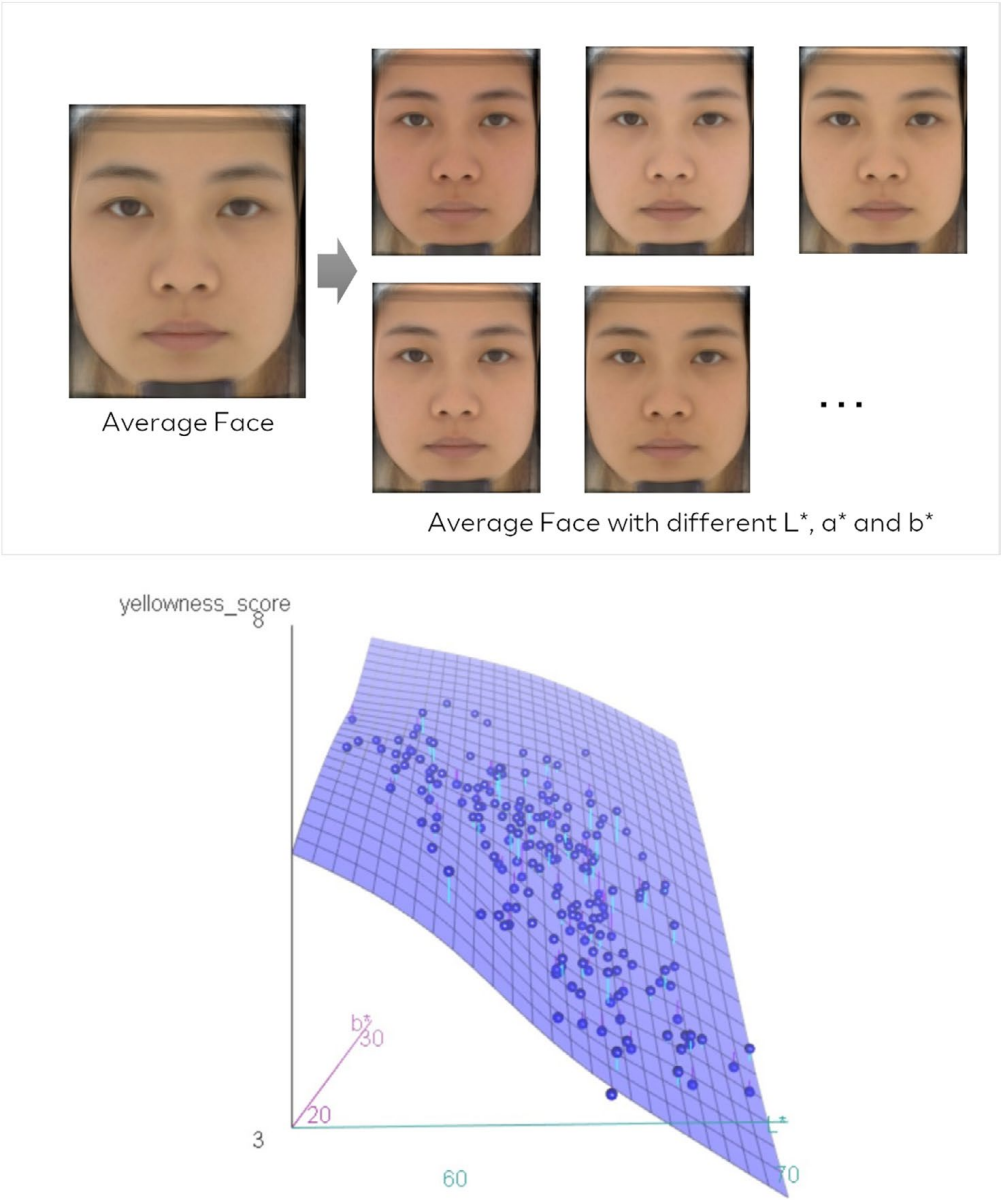
Demo of artificial faces from average face and distribution of b* and L* with perceptual yellowness score.

The above results indicated both colorimetric parameters and skin optical parameters could contribute to skin yellowness perception. In order to understand the contribution of the most important colorimetric parameters L* and b* to skin yellowness perception, observers were asked to attribute a yellowness score to 200 realistic artificial face images with colorimetric differences based on an average face picture generated by digital simulation. There is only colorimetric difference of the artificial face; different L*, a* and b* values were preset for these artificial face pictures, without any change of the face shape or skin texture information, which means only the colorimetric parameters could influence perception of these facial pictures as shown in Figure 3. The correlation of yellowness score and L*, a* and b* values by multiple linear regression analysis showed that L* is negatively correlated with yellowness perception, b* is positively correlated with yellowness perception, while a* value showed quite low correlation (*R* ^2^ = 0.21) with yellowness perception, which is consistent with previous findings in paired tests. With the multi linear regression model, the relation could be represented as Yellowness score = 0.42b* − 0.2L* + 9.01 (*R* ^2^ = 0.957). Besides, regression analysis was also used to define the contribution of each factor. It was shown that the contribution of L* to yellowness perception was 42.07%, while b* showed a higher contribution (57.92%).

### 3.3 Impact of Sun Exposure to Skin Yellowness Perception

**FIGURE 4:**
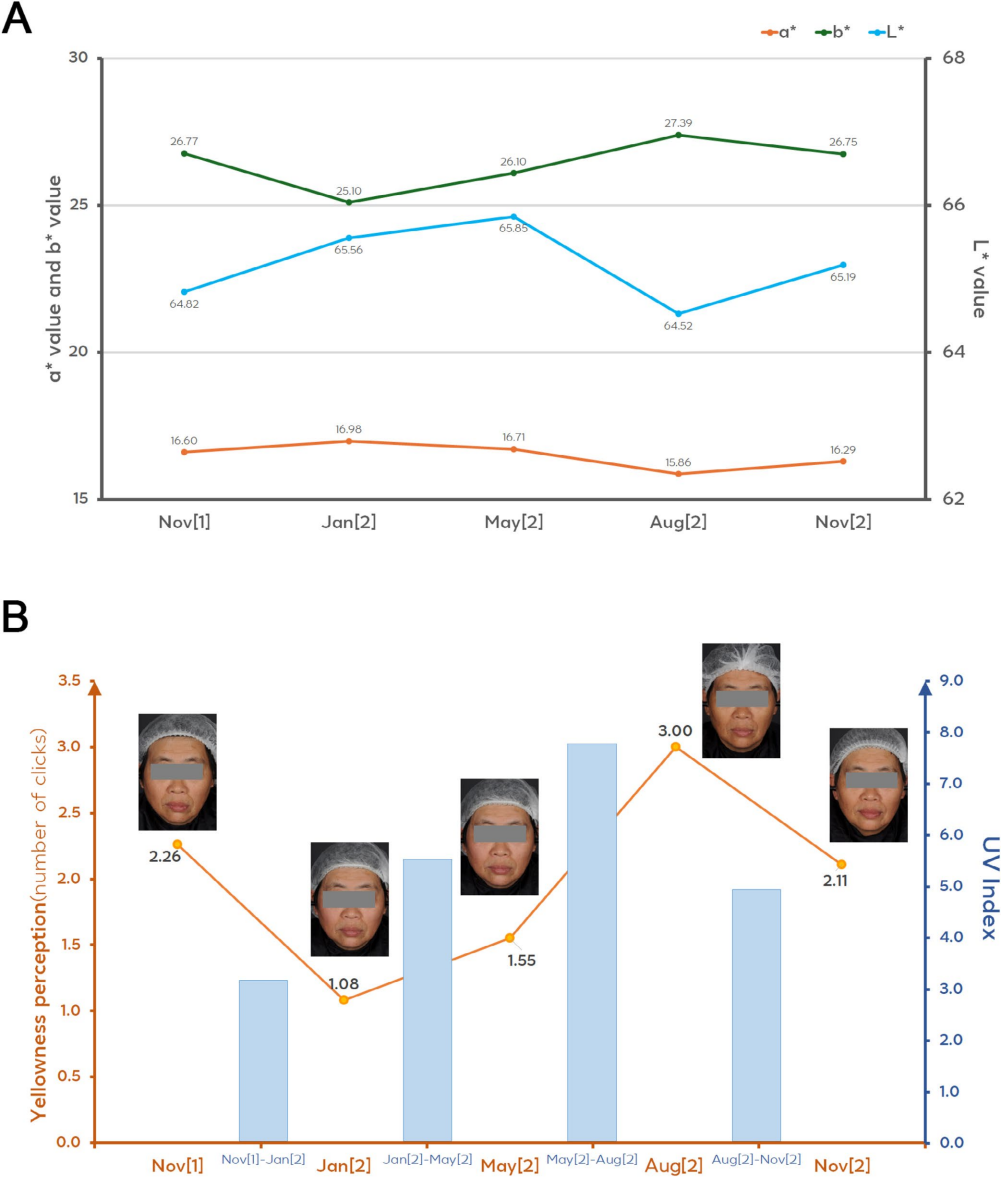
(A) Average evolution of L*, a* and b* during the year. Each instrumental evaluation time point was marked with the month. Nov[1] indicates November of the first year, Jan[2] indicates January of the second year, and the identification of other months follows the same rule thereafter. For L*, there is significant difference (*p* < 0.05) between each time point, except Nov[1] and Nov[2], Jan[2] and May[2]. For a*, there is significant difference (*p* < 0.05) between each time point, except Nov[1] and Jan[2], Nov[1] and May[2], Jan[2] and May[2]. For b*, there is significant difference (*p* < 0.05) between each time point, except Nov[1] and Nov[2]. (B) Skin yellowness perception during the year. For skin yellowness perception, as indicated by the number of clicks, there is significant difference (*p* < 0.05) between each time point, except Nov[1] and Nov[2]. The blue bar indicated the average UV index.

Since sun exposure is an important external exposome factor for skin health and skin appearance, including skin tone, another pair test was conducted to understand the impact of sun exposure on skin yellowness perception. A group of facial pictures (270 pictures in total) of 54 Chinese females from a previous 1‐year study, including 5 pictures taken for each volunteer during the year, was put in the 2nd pair test. During the year, the volunteers were asked to keep their normal skincare routine and normal real‐life activities. Based on previous instrumental evaluation data of these volunteers, colorimetric parameters of these volunteers showed significant evolution during the year in real‐life conditions, L* and a* values significantly decreased from winter to summer, while b* significantly increased (*p* < 0.05), as shown in Figure 4A. Similarly, the pair test results showed that consumer perception of skin yellowness changed with UV exposure dynamics during the whole year. Skin yellowness perception in the same volunteer during the year is highly positively correlated with b* value change, while negatively correlated with skin lightness (L*), as well as ITA° change which is calculated from L* and b*.

Obviously, skin yellowness perception changes along the year showed the same dynamic as sun exposure changes as shown in Figure 4B, skin yellowness perception significantly increased (*p* < 0.05) on the facial pictures from January to September, especially from May to September, then decreased due to lower UV index after September, which indicated that sun exposure might be an important environmental exposome factor that contributes to perceived skin yellowness.

## 4 Discussion

Skin tone plays an important role in the perception of facial attractiveness, self‐satisfaction of beauty and even judgment of a healthy lifestyle or physiological conditions. In chromatics, it is known that along the b* axis, higher b* represents a color shift toward yellow. However, in real life, facial perception is much more complex; it is not clear whether b* is enough to determine perception of facial skin yellowness. In this study both skin colorimetric parameters and optical parameters were associated with the perception of skin yellowness from a third‐person perspective.

Since the surface of skin is not smooth and planar, surface or specular reflection reflects about 4%–7% of the incident light; over 90% of light reflection of skin is from diffused reflection, which is internal reflection of diffuse, back‐scattered radiation [25]. When incident light passes through the skin surface and into the skin, it is refracted and whereas the majority of it might be absorbed or scattered [25, 26], reflected light from deeper layers of skin will also be impacted by the structure and components of the epidermis, including stratum corneum as a translucent diffusive layer. Together with specular reflection, diffused reflection contributes to skin radiance, which is normally related to attractiveness and skin health from inside. With the result that diffused reflection of skin is negatively correlated with skin yellowness perception, the decreased skin diffused reflection in elder people may at least partially explain the increased impression of skin yellowness in elder people. Moreover, in a younger group of people, not only is diffused reflection but also specular reflection shown to be negatively correlated with skin yellowness perception, which indicates that for younger female skin smoothness or skin texture may also impact the perception of skin yellowness; smoother skin may give lower perceived yellowness.

The CIE system characterizes colors by luminance and color coordinates; it's commonly used to describe skin color in clinical evaluation. In this study by pair test and digital simulation‐rating test, it is shown that together with b*, L* is also an important parameter which determines perception of skin yellowness, with comparable contribution of b*, while the impact of a* is relatively limited on the perception of skin yellowness. Since it's known that skin lightness (L*) decreases while b* increases with age in Chinese female [6], together with our findings on perception, it is not hard to explain the increased skin yellowness concern with aging. L* is often associated with melanin index in clinical studies, which indicates that melanin quantity might be an important impact factor not only to skin lightness, but also to skin yellowness perception. By digital simulation and rating test, a formula was generated to present the correlation of yellowness perception and colorimetric parameters L* and b*; it could also provide the range to what extent a treatment effect should achieve to get a perceived efficacy of skin yellowness improvement. Especially, the observers for the quiz test are skin brightening product users, who might also represent the target population of a treatment for skin yellowness improvement, providing guiding significance at the application level for developing these treatments.

In previous clinical research, it has been reported that from winter to summer, along with UV exposure dynamics, several parameters regarding skin pigmentation were significantly altered, including decreased L* and increased melanin index, which was identified as “seasonal skin darkening” [27]. In this study, with the pair test on 1‐year tracking facial images, it was demonstrated that the perception of skin yellowness also changes along the year with an increase from winter to summer, which is consistent with changes in skin colorimetric parameters and UV exposure, indicating a perceivable “seasonal skin yellowing.” Considering the undeniable impact of sun exposure, it may further emphasize the importance of photoprotection in protecting skin from skin tone problems. Undeniably, there are also other external exposome factors, such as pollution or lifestyle changes, that might influence skin tone throughout the year; further exploration on the mechanism from exposome factors to skin colorimetric and optical parameters is necessary for a complete understanding of perceivable “seasonal skin yellowing.”

The present study disclosed the correlation of yellowness perception and facial skin clinical parameters, including both colorimetric parameters and optical parameters, especially the contribution proportion of relevant colorimetric parameters. In previous research, b* was used as the only parameter to represent “yellowness,” in the present study, from a perception view, b*, L* and diffused reflection are all important parameters associated with perceived skin yellowness for both young and elder Chinese females. Further, the perception test indicated that for younger female skin surface property may also impact the perception of skin yellowness, as shown by the correlation with specular reflection. These findings provided new insights for cosmetics development targeting skin aging yellowness, photoprotection and modulation of skin parameters including L*, b*, reflection could be considered as clinical targets to improve perception of yellowness. Skin yellowness is also a perception of the individual; it's difficult to assess it through a single objective parameter, Now, for cosmetic products development and efficacy validation, it could be assessed through combined objective parameters such as L* and b*. Furthermore, this study allows us to define thresholds of these parameters for cosmetic products to reach perceived efficacy. Future research on the contribution of quantified optical parameters could complete the assessment to a greater extent.

Besides, the biological mechanism underlying these skin parameters changes with age is still unclear; further exploration on skin biology will be critical to find the biological alterations during skin aging in correlation with clinical parameters of skin yellowness, which could help to identify biological targets and provide skincare solutions that work through biological regulation.

## Author Contributions

Ping Wang, Jie Qiu and Juanjuan Chen contributed to the conceptualization of this research. Jie Qiu managed the research. Juanjuan Chen, Chengda Ye and Virginie Hourblin designed the test protocols. Yang Wang conducted the tests. Etienne Huguet and Emmanuelle Mainguene contributed to the data interpretation. Ping Wang wrote the paper and Frederic Flament reviewed the paper.

## Ethics Statement

All participants were provided with a detailed explanation of the research objectives and procedures; written informed consent was obtained accordingly. All personal data were anonymized, and confidentiality was strictly maintained. This study was conducted in accordance with the ethical standards of the institutional and national guidance.

## Conflicts of Interest

All the authors are employees of L'Oréal Group. The authors declare no conflicts of interest.

## Data Availability

The data that support the findings of this study are available from the corresponding author upon reasonable request.
